# Direct binding to GABARAP family members is essential for HIV-1 Nef plasma membrane localization

**DOI:** 10.1038/s41598-017-06319-4

**Published:** 2017-07-20

**Authors:** Alexandra Boeske, Melanie Schwarten, Peixiang Ma, Markus Tusche, Jessica Mötter, Christina Möller, Philipp Neudecker, Silke Hoffmann, Dieter Willbold

**Affiliations:** 1 Institute of Complex Systems, Structural Biochemistry (ICS-6), Forschungszentrum Jülich, 52425 Jülich, Germany; 2 Institut für Physikalische Biologie, Heinrich-Heine-Universität Düsseldorf, 40225 Düsseldorf, Germany; 3 Present Address: Shanghai Institute for Advanced Chemical Studies (SIAIS) ShanghaiTech University, Shanghai, 201012 China

## Abstract

HIV-1 Nef is an important pathogenic factor for HIV/AIDS pathogenesis. Studies have shown that the association of Nef with the inner leaflet of the plasma membrane and with endocytic and perinuclear vesicles is essential for most activities of Nef. Using purified recombinant proteins in pull-down assays and by co-immunoprecipitation assays we demonstrate that Nef binds directly and specifically to all GABARAP family members, but not to LC3 family members. Based on nuclear magnetic resonance (NMR) experiments we showed that Nef binds to GABARAP via two surface exposed hydrophobic pockets. S53 and F62 of GABARAP were identified as key residues for the interaction with Nef. During live-cell fluorescence microscopy an accumulation of Nef and all GABARAP family members in vesicular structures throughout the cytoplasm and at the plasma membrane was observed. This plasma membrane accumulation was significantly reduced after knocking down GABARAP, GABARAPL1 and GABARAPL2 with respective siRNAs. We identified GABARAPs as the first known direct interaction partners of Nef that are essential for its plasma membrane localization.

## Introduction

The human immunodeficiency virus (HIV) boosts its replication and eludes host defense mechanisms by manipulating the molecular machinery of the host cell, especially protein trafficking pathways within the endomembrane system. Most of these activities are accomplished by the accessory proteins of HIV, which are not essential for virus replication *in vitro*, but play a critical role in developing a cellular environment that is optimal for viral replication and spread *in vivo* ^1^.

One of the first and most abundantly expressed proteins during the early stages of viral infection is the accessory protein Nef (negative factor)^2^. Nef is even contained in HIV particles when infecting the cell^3^, is a 27 to 35 kDa non-enzymatic protein and is encoded by HIV-1, HIV-2 and SIV^4^. The three-dimensional structure of Nef has been investigated by X-ray crystallography^5, 6^ and nuclear magnetic resonance (NMR) spectroscopy^7, 8^. The overall three-dimensional organization of the HIV-1 Nef protein is governed by three distinct structural entities: the largely unstructured N-terminal anchor domain (residues 1–61 in the case of Nef from HIV-1_SF2_), a well-folded core domain (62–210), and a long flexible loop (152–184)^9^. The unstructured flexible regions in particular form a large and variable surface that may be essential for the numerous interactions of Nef with host cell proteins^10^. One well described function of Nef is the expression level control of various cell surface receptors that play important roles in immunity and the virus life cycle by interfering with the itinerary of proteins within the endocytic and late secretory pathways^1, 11^. Association of Nef with the host cell membrane, especially with the inner leaflet of the plasma membrane but also with endocytic and perinuclear vesicles, appears to be advantageous in fulfilling these functions^9, 12–16^. Membrane association by Nef is assisted by cotranslational myristoylation at residue G2, which is N-terminally exposed after the N-terminal methionine is cleaved. Together with an arginine rich sequence (R17/19/21/22) the myristoyl group forms an N-terminal membrane targeting module^9^. Although this N-terminal domain was characterized as a key determinant for overall membrane association, it remains unclear how specific targeting of Nef to and association with the plasma membrane is achieved^17^.

Autophagy-related 8 (Atg8) proteins form a highly conserved protein family specific to eukaryotes^18^. Yeast has a single Atg8 protein, whereas in humans several ATG8 family members (later referred to as ATG8s) exist, which are divided in two subfamilies. The microtubule-associated protein 1 light chain 3 (MAP1LC3; referred to as LC3) subfamily includes three members LC3A, LC3B and LC3C (later referred to as LC3s), and the GABARAP/GATE-16 subfamily includes γ-amino-butyric acid receptor-associated protein (GABARAP), Glandular epithelial cell protein/GABARAP-like 1 (GEC1/GABARAPL1) and Golgi-associated ATPase enhancer of 16 kDa/GABARAP-like 2 (GATE-16/GABARAPL2) (later referred to as GABARAPs). ATG8 proteins play important roles in autophagy and undergo lipidation mediated by a ubiquitin-like conjugation system during this event^19^.

Autophagy is an evolutionarily highly conserved process that is essential for cellular homeostasis under both normal and stress conditions, such as nutrient deficiency. This dynamic process ensures survival of cells by degrading cellular proteins and even whole cell organelles^20, 21^. In this process, portions of the cytoplasm are engulfed into double-membrane vesicles, the so-called autophagosomes, to deliver their contents to the lysosome for degradation. All mammalian (m)ATG8s share a high structural similarity. The tertiary structures consist of an ubiquitin-like fold with two additional α-helices located N-terminally. This arrangement exposes two hydrophobic pockets, which are conserved among family members and mediate protein-protein interactions. These interactions occur primarily via recognition of a conserved motif termed the LC3-interacting region (LIR) or the Atg8-interacting motif (AIM)^22, 23^. During autophagy mATG8s fulfill three functions: (i) they mediate expansion and closure of the phagophore membrane; (ii) they act as cargo receptors that recruit cytoplasmic cargo through the LIR motif; and (iii) they serve as adaptor proteins that recruit signaling and trafficking proteins as well as the autophagy machinery to the autophagosome. In addition, a growing number of studies describes specific autophagic and additionally non-autophagic functions of each ATG8^18, 24–28^. Intriguingly, as they were originally described to be involved in intracellular transport of the GABA type-A receptor^29^, GABARAPs appear to generally facilitate transport and recruitment of membrane-bound factors^30–33^.

In a previous study we identified a panel of putative HIV-1 Nef interacting partners by a membrane-associated yeast two-hybrid (Y2H) screen^34^. Using an unrelated control bait, specific Nef binding (“bait-dependency”) was not shown unambiguously for 37 of the 58 hits, including GABARAPL2, and thus, these proteins were not reported in the published hit list. Encouraged by the increasing number of publications dealing with the intersection of HIV propagation and autophagy^35^ together with the identification of GABARAPL2 and GABARAP as proteins relevant for HIV infection/replication in two independent, large scale siRNA screens^36, 37^, we decided to have a more detailed look at the interaction between Nef and the human ATG8s with the aim of uncovering the putative biological function of this cross-talk, which might be relevant e.g. for Nef-associated pathogenesis during HIV infection. The underlying interaction was validated by independent methods including coimmunoprecipitation (CoIP) in mammalian cell culture and pulldown analysis using purified recombinant proteins. Both GABARAP and LC3B family members as wildtype or chimeric mutants were employed in our analysis to delineate the selectivity of Nef. Mapping the Nef binding site onto the GABARAP surface by NMR spectroscopy allowed a comparison with the binding site of canonical ligands. Finally, live cell imaging using fluorescent protein tagged Nef and GABARAP, partially combined with GABARAPs silencing by siRNA, let us realize that the anterograde transport of Nef, a suggested prerequisite for Nef-mediated pathogenesis, is dependent on the activity of GABARAP.

## Results

### Nef immunoprecipitates with GABARAPs, but not with LC3B, in mammalian cell extracts

**Figure 1:**
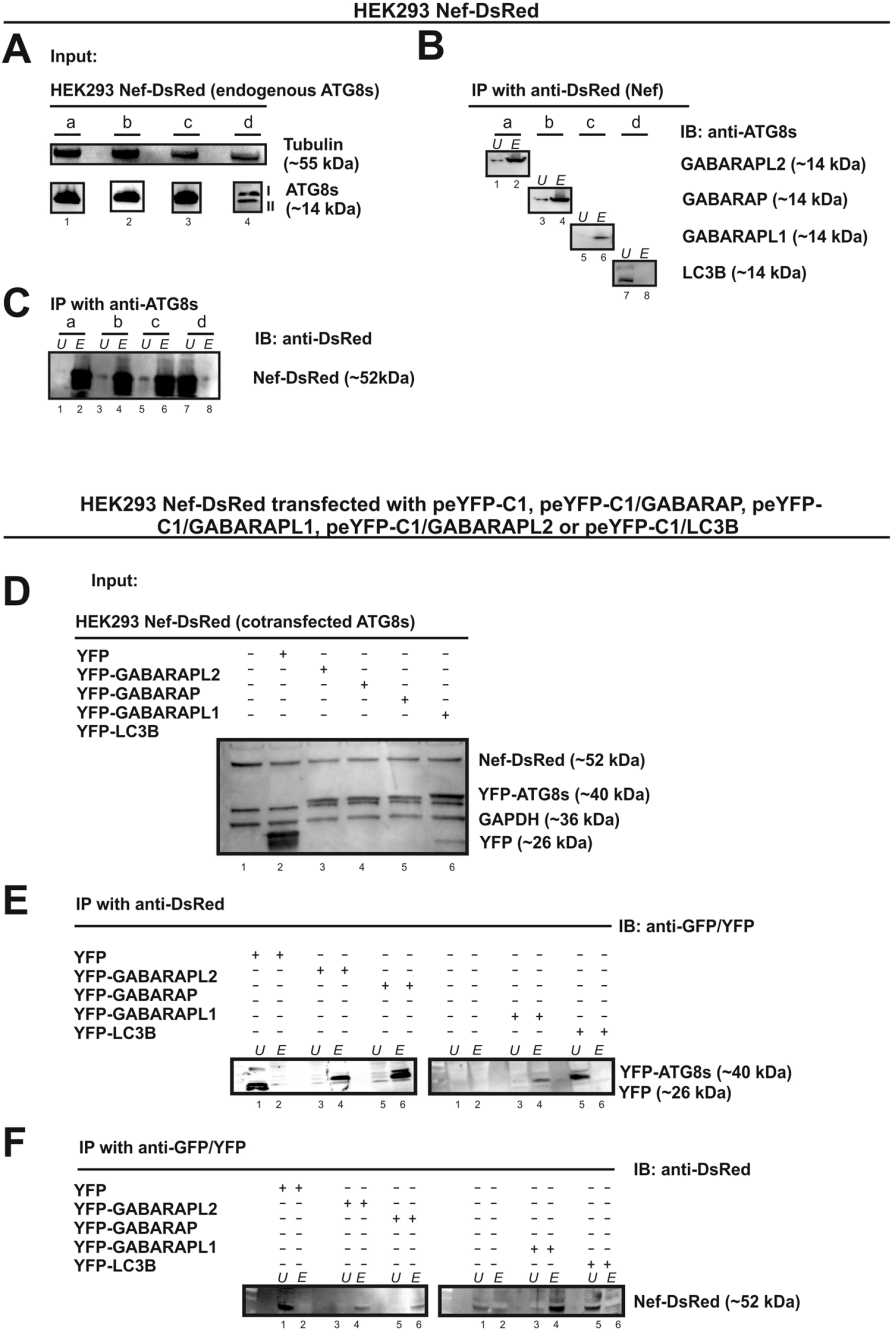
Co-immunoprecipitation (IP) of the Nef-ATG8 complex. Co-IP studies were carried out with lysates prepared from HEK293 cells stably expressing Nef-DsRed (**A–C**) and cotransfected with plasmids encoding for the indicated YFP-ATG8 constructs (**D–F**). (**A**) and (**D**) show samples before immunoprecipitation. In (**A**) an anti-GABARAPL2 (a), an anti-GABARAP (b), an anti-GABARAPL1 (c), and an anti-LC3B antibody (d) were used to detect the different ATG8s. (**B**) Immunoprecipitates with anti-DsRed antibody followed by SDS-PAGE and immunoblotting with various ATG8 antibodies (a: anti-GABARAPL2, b: anti-GABARAP, c: anti-GABARAPL1 and d: anti-LC3B). (**C**) In a reciprocal set of experiments, immunoprecipitates were carried out with various ATG8 (a: anti-GABARAPL2, b: anti-GABARAP, c: anti-GABARAPL1 and d: anti-LC3B) antibodies followed by SDS-PAGE and immunoblotting with an anti-DsRed antibody. In (**D**) an anti-DsRed antibody was used to detect Nef fused to DsRed and an anti-YFP antibody to detect YFP and the ATG8s fused to YFP. (**E**) Immunoprecipitates with an anti-DsRed antibody or (**F**) with an anti-GFP antibody followed by SDS-PAGE and immunoblotting with the antibodies are indicated. Data are representative of three independent experiments (U: unbound material; E: eluate fraction). Full-length western blots are presented in Supplementary Figs 1 and 2.

Initially, CoIP of GABARAPs and also LC3B with Nef was examined. For this set of experiments lysates from HEK293 cells stably transfected with pDsRed-N1/Nef were subjected to immunoprecipitation (IP) and immunoblotting (Fig. 1A–C). The expression levels of Nef-DsRed and endogenous ATG8s in the indicated lysates were determined (Fig. 1A), before incubating the lysate with anti-GABARAP, -GABARAPL1, -GABARAPL2 and -LC3B antibodies. Nef was found to co-immunoprecipitate with GABARAP, GABARAPL1 and GABARAPL2 (Fig. 1B), but not with LC3B. Reverse IPs were performed with each ATG8 and consistently revealed the presence of Nef in the protein complexes upon GABARAP, GABARAPL1 and GABARAPL2 precipitation (Fig. 1C). LC3B did not precipitate with Nef. Since antibody specificity of the commercially available antibodies has been doubted^38^, we additionally performed CoIPs in a different experimental setting. Thus, HEK293 cells stably transfected with pDsRed-N1/Nef were transfected with peYFP-C1, peYFP-C1/GABARAP, peYFP-C1/GABARAPL1 and peYFP-C1/GABARAPL2. The resulting lysates were subjected to anti-DsRed or anti-YFP IP and immunoblotting with anti-YFP or anti-DsRed antibody, respectively (Fig. 1D–F).

Nef precipitated via its DsRed moiety revealed the presence of YFP-GABARAP, YFP-GABARAPL2 and YFP-GABARAPL1, but not that of YFP-LC3B and YFP in the precipitates (Fig. 1E). The reciprocal experiment resulted in the presence of Nef-DsRed in the immunoprecipitates generated with the anti-YFP antibody when using the extracts from YFP-GABARAP, YFP-GABARAPL2 and YFP-GABARAPL1 expressing cells, but not with extracts of YFP-LC3B or YFP expressing cells (Fig. 1F).

### Nef directly binds to GABARAP, GABARAPL1 and GABARAPL2

**Figure 2:**
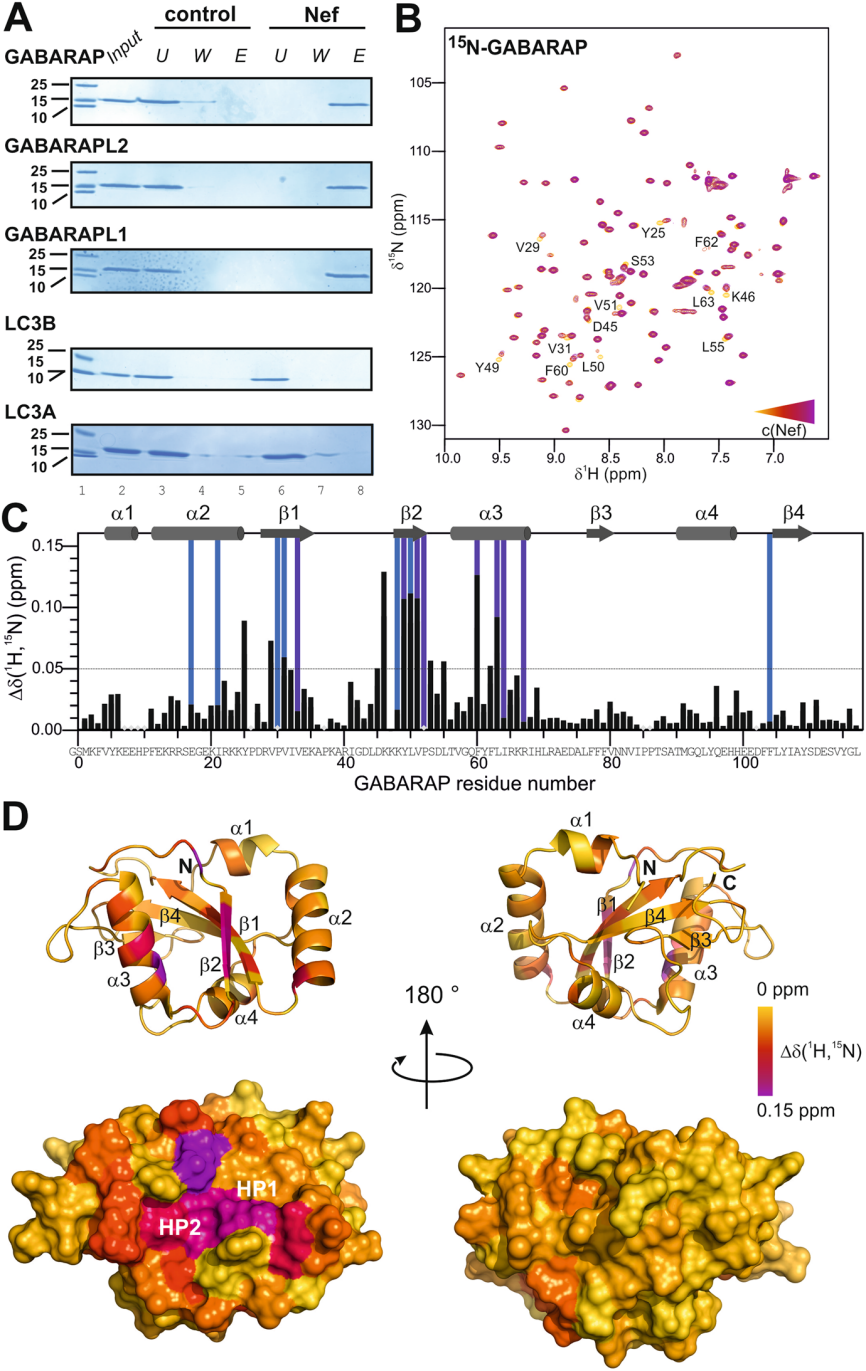
Nef selectively binds to GABARAPs in a direct manner and contacts the canonical ligand binding site of GABARAP. (**A**) Nef-conjugated or free control Sepharose beads were incubated with the purified recombinant ATG8 paralogs listed. The input, the unbound material of the flow through (U), the wash (W) fractions and the eluate (E) fractions have been analyzed by SDS-PAGE and CBB staining. Full-length gels are presented in Supplementary Fig. 4. (**B**) Mapping the Nef binding site on GABARAP by NMR titration experiments: overlay of 2D ^1^H-^15^N-HSQC spectra of [*U*-^15^N]-GABARAP (yellow) with increasing amounts of Nef (red and purple). GABARAP residues affected upon Nef binding are labelled. For better visibility the first titration step (ratio of 1:0.5 for [GABARAP]:[Nef]) has been omitted from this figure. (**C**) Combined chemical shift changes Δδ(^1^H,^15^N) as a function of the GABARAP sequence. Secondary structure elements are shown on top of the diagram. Residues belonging to the hydrophobic pockets 1 and 2 are highlighted in light blue and blue, respectively. Residues for which no chemical shift changes could be determined and prolines are marked by a grey diamond. A cut-off of 0.05 ppm (continuous horizontal line) was used for significance. (**D**) Mapping of the Nef interaction site on a schematic representation of the secondary structure (top) and the molecular surface (bottom) of GABARAP (PDB ID: 1KOT). Residues strongly affected upon Nef binding are marked in purple, whereas non-interacting residues are shown in yellow.

To clarify whether the observed association of Nef with GABARAP, GABARAPL1 and GABARAPL2 is direct or mediated by yet unknown intermediate partners, we prepared recombinant human ATG8s and recombinant Nef protein purified to almost complete homogeneity (Supplementary Fig. 3), and subjected them to direct *in vitro* binding assays. Nef protein was immobilized on Sepharose-beads via amine coupling, and the resulting Nef-Sepharose was exposed to each of the purified ATG8s. After extensive washing bound proteins were eluted, precipitated, separated by SDS-PAGE, and CBB stained. Clearly GABARAP, GABARAPL1 and GABARAPL2 (Fig. 2A) were retained by the Nef-coupled Sepharose, but not by the Sepharose without immobilized Nef protein, confirming that the interaction between Nef and GABARAPs is direct and does not need an additional factor. Consistent with the immunoprecipitation results no protein was obtained in the eluate fractions for LC3B. Additionally, another LC3 subfamily member, LC3A, could not be retained by Nef in this assay (Fig. 2A).

### The canonical hydrophobic ligand binding pockets of GABARAP are involved in direct Nef binding

Since there is high sequence conservation between the GABARAPs (GABARAP with GABARAPL1: 87% identity and 97% similarity; GABARAP with GABARAPL2: 57% identity and 97% similarity), we hypothesized that the three GABARAPs display a common interface for Nef binding. GABARAP was chosen for molecular level investigation because this protein is the most structurally characterized member of this subfamily^39–49^. To identify GABARAP residues that are involved in Nef binding, two-dimensional (2D) ^1^H-^15^N-heteronuclear single quantum coherence (HSQC) spectra of ^15^N-GABARAP were recorded during titration with unlabeled Nef protein (Fig. 2B). The spectrum without Nef exhibited the typical signals of natively folded GABARAP^44^. Addition of Nef caused chemical shift changes to several resonances, demonstrating that the proteins interact. As depicted in Fig. 2C, these resonance signals correspond, among others, to amino acid residues V31 and L50, and to residues Y49, V51, F60 and L63, which are part of the canonical hydrophobic pockets 1 (HP1) and 2 (HP2), respectively. HP1 and HP2 have been described to be conserved binding sites for various ligands of GABARAP^50, 51^. In addition, Y25, V29, D45, K46, S53, L55 and F62, which are residues adjacent to HP1 and HP2, were affected upon Nef binding. Representing the affected residues on the surface of GABARAP, the Nef-binding region mapped on a contiguous region similar to that known for other GABARAP ligands, and mainly consisted of three linker regions (α2-β1, β1-β2, β2-α3), β-strands β1 and β2, as well as helix α3, whereby residues within helix α2 show only minor chemical shift changes. HP1 seemed to be less affected than HP2 (Fig. 2D).

Because the NMR titration experiments were carried out at concentrations well above the dissociation constant, any determination of the dissociation constant from the chemical shift titration data could lead to an overestimation of the dissociation constant. Therefore, we used surface plasmon resonance to determine an estimate of the affinity of Nef and GABARAP *in vitro* and obtained a *K*_*d*_ value of about 20 µM (Supplementary Fig. 5), which is in the range of known GABARAP-ligand interactions^40^.

### Conserved residues among the GABARAPs are key residues for Nef binding

**Figure 3:**
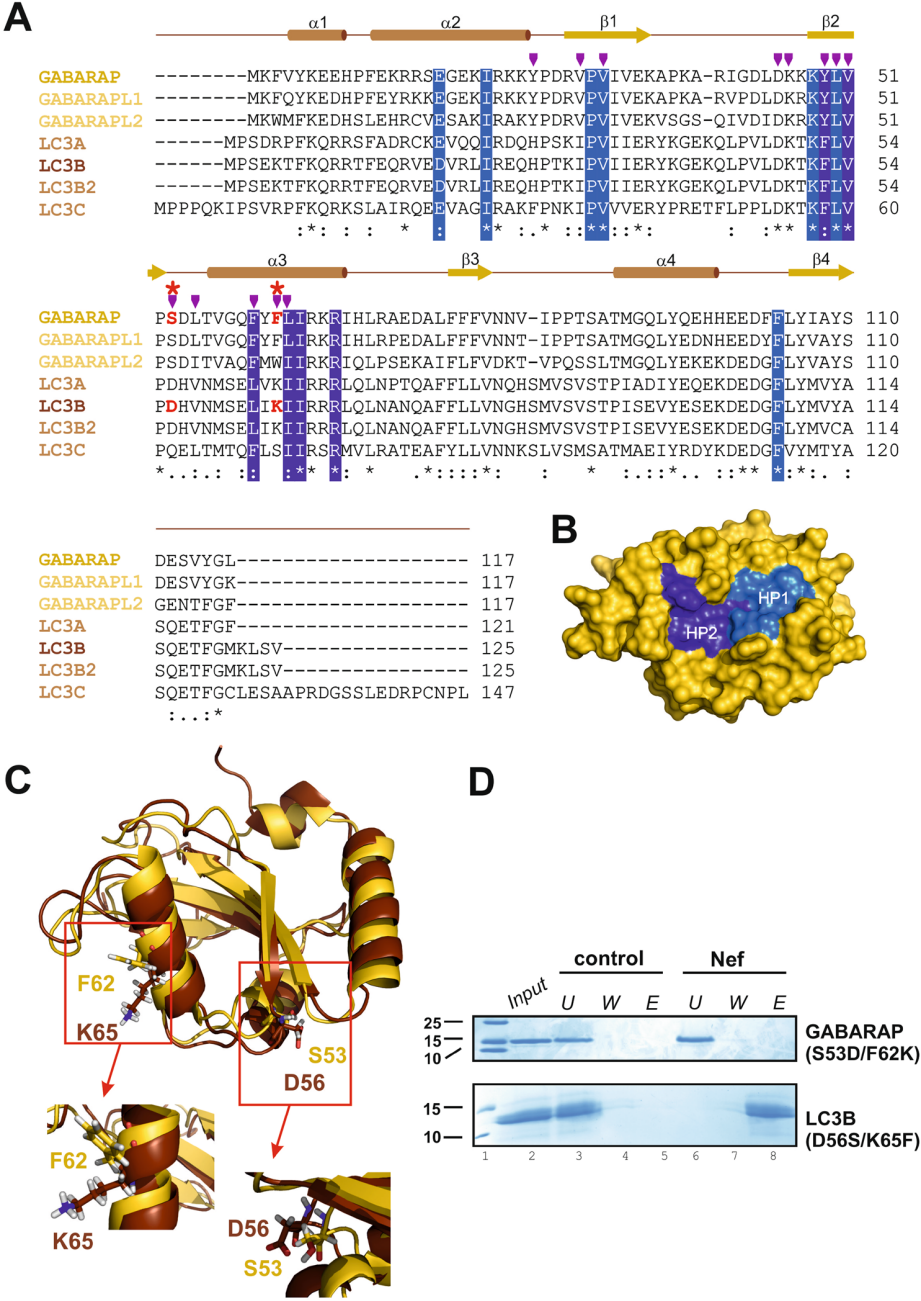
GABARAP residues S53 and S62 are essential for Nef binding. (**A**) Human ATG8s sequence alignment indicates putative key residues for Nef binding specificity. Protein names of ATG8s showing Nef binding during pull-down and immunoprecipitation analysis are given in different shades of yellow, and include all members of the GABARAP subfamily. Protein names of LC3B and of the other LC3s are given in different shades of brown. Purple arrows indicate residues of GABARAP showing chemical shift changes higher than 0.05 ppm (see Fig. 2C) upon Nef titration. Residues forming HP1 and HP2 are shaded in light and dark blue, respectively. Red asterisks highlight putative key positions for determining Nef-binding specificity. The corresponding amino acids found in GABARAP and LC3B at these positions are highlighted in red. (**B**) Visualization of HP1 and HP2 on the surface of the GABARAP structure [PDB ID: 1KOT]. (**C**) Structural overlay of GABARAP and LC3B with the putative key residues necessary for Nef-binding highlighted. Cartoon representations of GABARAP (yellow) and LC3B (brown) [PDB ID: 1V49] showing their regular secondary structure elements demonstrate that S53 and F62 of GABARAP as well as the corresponding D56 and K65 of LC3B are surface exposed, and therefore are available for ligand binding. Details highlight the side chain orientations of the key residues, for this the structures have been rotated appropriately. (**D**) Nef-conjugated or free Sepharose beads (control) were incubated with GABARAP(S53D/F62K) or LC3B(D56S/K65F) mutants. The input, the unbound material of the flow through (U),the wash (W) fractions and the eluate (E) fractions were subjected to SDS-PAGE and visualized by CBB staining. Full-length gels are presented in Supplementary Fig. 7.

Nef interacts with GABARAP primarily through the two hydrophobic binding pockets on the surface of the GABARAP protein (Fig. 2D). These hydrophobic binding pockets, that form a canonical ligand docking site, represent the conserved binding sites for a diverse range of ligands of GABARAP. To understand why Nef interacts with GABARAP, GABARAPL1 and GABARAPL2, but not with LC3s, the NMR chemical shift mapping data obtained for the GABARAP:Nef complex was combined with the results of our interaction studies and with a multiple sequence alignment of the mATG8s (Fig. 3A). This analysis revealed that residues comprising the core of the two hydrophobic ligand binding pockets are generally well conserved between all mATG8s. Residues affected upon Nef binding that do not correspond to the core binding pockets comprise conserved ones as well as residues that are only conserved within GABARAPs. More specifically residues D45 and K46 are identical and V29 of GABARAPs is similar to I31 of LC3s. Y25, which has been previously shown to be important for specific binding of ALFY to the GABARAP family^52^, is exchanged in LC3A and LC3B to the equally aromatic H27 or the aromatic F33 of LC3C. In contrast, S53 and F62 in GABARAP are clearly influenced upon Nef titration, and show conservation only between the GABARAPs but not with the LC3s. This suggests that these surface exposed residues, which are located immediately following the second β-strand or reside in the middle of helix α3, respectively (Fig. 3C), might be responsible for the observed selective binding of Nef to GABARAPs.

### Residues S53 and F62 of GABARAPs determine their binding specificity for Nef and are sufficient for transferring Nef binding to LC3

To prove the importance of serine and phenylalanine at positions 53 and 62, respectively, we substituted these GABARAP residues with aspartate and lysine, which can be found at the corresponding positions in LC3B (Fig. 3A). Since the inability to interact with a certain binding partner observed after mutation could also be caused by a general loss of structure, we first analyzed the secondary structure of the obtained GABARAP(S53D/F62K) mutant by circular dichroism (CD) spectroscopy. The CD spectra were identical between the mutant and wild type proteins, indicating that the overall secondary structure was near identical (Supplementary Fig. 6A). In addition, a 2D ^1^H-^15^N-HSQC spectrum of ^15^N-GABARAP (S53D/F62K) was recorded and compared with the 2D ^1^H-^15^N-HSQC spectrum of the wild-type protein (Fig. 2B). Superposition of the spectra shows that both proteins have very virtually identical folds (Supplementary Fig. 6B). When analyzed for its Nef binding capability this GABARAP mutant could not be retained on Nef-Sepharose any more (Fig. 3D), supporting an essential role of both or at least one of the residues probed. Next, we wanted to know whether the corresponding substitution of LC3B residues D56 and K65 with the corresponding GABARAP residues S53 and F62 would be sufficient to establish Nef-binding activity in the otherwise Nef binding inactive LC3B. Again, we first confirmed the structural integrity of the corresponding LC3B (D56S/K65F) mutant protein as described above for the GABARAP mutant (Supplementary Fig. 6C and D). Retention of the LC3B (D56S/K65F) mutant on Nef-coupled Sepharose (Fig. 3D) showed that exchange of these two residues was sufficient to establish Nef binding activity of LC3B.

### GABARAP, GABARAPL1 and GABARAPL2 colocalize with Nef in vesicular structures

**Figure 4:**
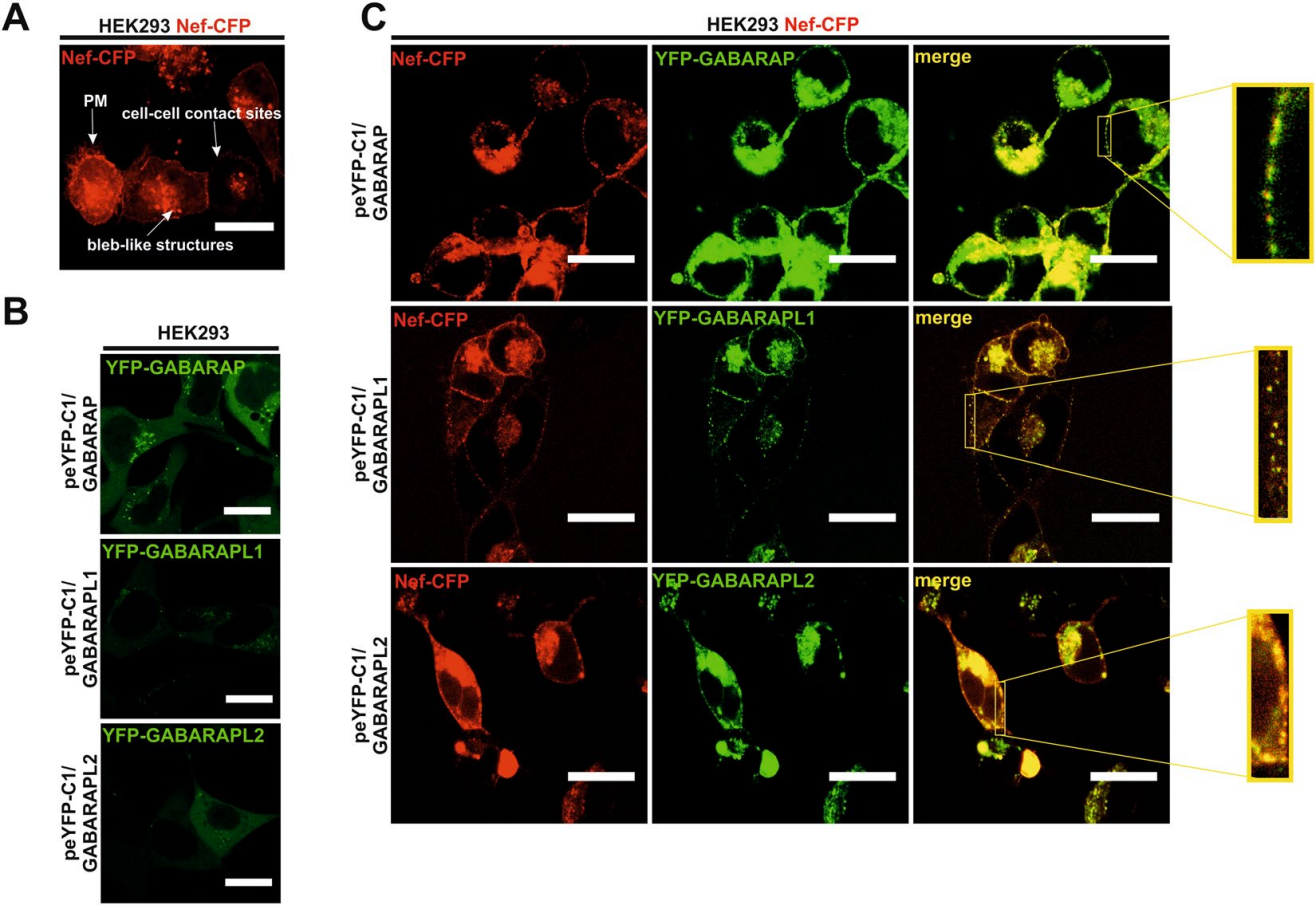
GABARAP, GABARAPL1 and GABARAPL2 colocalize with Nef in vesicular structures. (**A**) Live-cell fluorescence microscopy of HEK293 cells stably expressing Nef-CFP. Here, Nef was observed at cell-cell contact sites, bleb-like structures or at the plasma membrane (PM). Scale bar 20 µm. (**B**) Live-cell fluorescence microscopy of HEK293 cells transfected with peFP-C1/GABARAP (top), peYFP-C1/GABARAPL1 (middle) and peYFP-C1/GABARAPL2 (bottom) revealed a diffuse distribution pattern for all GABARAPs. Scale bar 20 µm. (**C**) Live-cell fluorescence microscopy of HEK293 cells stably expressing Nef-CFP (red) and transfected with peFP-C1/GABARAP (top), peYFP-C1/GABARAPL1 (middle) and peYFP-C1/GABARAPL2 (bottom) (all green). In this case, Nef and GABARAPs showed high colocalization levels at vesicular structures and at the plasma membrane (see merge and zoom). Scale bar 20 µm.

In order to explain the role of the direct interaction of Nef with GABARAPs, we investigated the localization of Nef and GABARAPs in cells. ATG8s were originally implicated in membrane trafficking processes^53^. GABARAP family members facilitate transport of vesicular structures along microtubules^54, 55^ to the plasma membrane, and here mediate the fusion of these vesicles with the plasma membrane^56–58^. Anticipating that GABARAPs are involved in the trafficking of Nef to the plasma membrane, we investigated whether Nef localizes with the GABARAPs in vesicular structures and whether expression of Nef and the ATG8 proteins lead to changes in their distribution patterns when compared with that of the patterns obtained for the individual factors. As a basis for comparison, we first analyzed the distribution pattern of Nef in stably peCFP-N1/Nef transfected cells. Here we observed a distribution pattern for Nef similar to the situation in Jurkat T-cells^59^. Nef was found at vesicular structures that were distributed between the Golgi region and at the well-stained plasma membrane (Fig. 4A). We next analyzed the distribution patterns of YFP-GABARAP, -L1 and –L2 in the absence of Nef using HEK293 cells transfected with plasmids coding for each of the YFP-GABARAPs by live-cell fluorescence microscopy 48 h post-transfection. GABARAP, GABARAPL1 and GABARAPL2 showed diffuse patterns of distribution, partially in puncta, throughout the cytoplasm (Fig. 4B). Finally, HEK293 cells constitutively expressing Nef-CFP were transfected with plasmids coding either for YFP-GABARAP, YFP-GABARAPL1 and YFP-GABARAPL2. Respectively, these cells revealed high colocalization levels of every of the GABARAPs with Nef (Fig. 4C). Compared with the mainly cytoplasmic distribution of the GABARAPs when overexpressed in the absence of Nef (Fig. 4B), the bulk of the GABARAPs localized together with Nef at membranous compartments such as the periphery of the cell, and here, predominantly at concentrated vesicular structures, the plasma membrane (see zoom) or inside tubular cell-cell contact sites, thus following the Nef pattern observed in cells overexpressing Nef-CFP alone (Fig. 4C).

### Small interfering RNA-mediated knockdown of GABARAPs reduces plasma membrane localization of Nef

**Figure 5:**
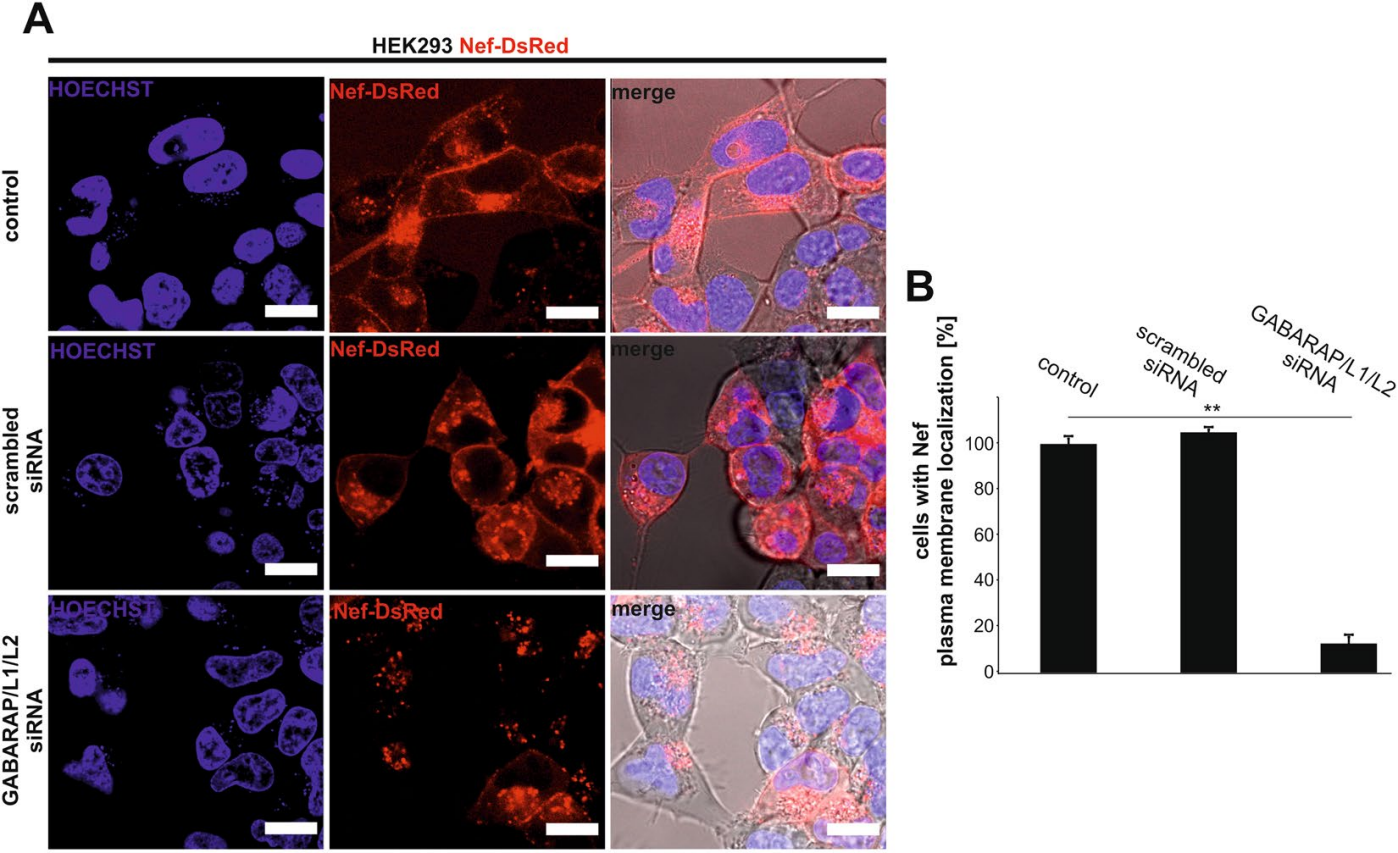
GABARAPs are required for plasma membrane localization of Nef. (**A**) Live-cell fluorescence microscopy of HEK293 cells stably expressing Nef-DsRed (red) and mock transfected as a control (top), transfected with scrambled siRNA (middle) or with a pool of GABARAP, -L1 and –L2 siRNAs (bottom). Hoechst 33442 was used to stain the nucleus (blue). Scale bar 20 µm. (**B**) Quantification of cells with Nef plasma membrane localization after treatment with the respective siRNAs from three independent experiments. **p < 0.05. The average value of three control experiments was set to 100%.

Finally, we wanted to investigate, whether a lack of GABARAPs has any effects on the subcellular distribution pattern of Nef. Therefore, we transfected Nef-DsRed expressing cells with different pools of siRNAs against GABARAP, GABARAPL1 and GABARAPL2 or with a scrambled control siRNA pool. Seventy-two hours post transfection live cell imaging was done with stably Nef-DsRed expressing cells that were untreated, transfected with scrambled siRNA or with a mixture of siRNAs against GABARAPs. The respective confocal fluorescence microscopy images were analyzed for the plasma membrane localization of Nef (Fig. 5A). In cells silenced for all GABARAPs Nef accumulated in many intracellular punctate structures and showed a drastically impaired Nef localization at the plasma membrane, when compared with that of Nef expressing cells in the presence of endogenous GABARAPs. In addition, no bleb-like structures were visible upon GABARAPs knockdown. To estimate the effect of the knockdown of GABARAPs on the plasma membrane localization we counted the cells in all samples by distinguishing between cells with and without Nef plasma membrane staining and analyzed them statistically. While transfection of control siRNAs had no significant effect on the percentage of cells showing plasma membrane localization of Nef, knockdown of all GABARAPs significantly decreased the number of cells harboring Nef at the plasma membrane to less than 20% of the value obtained for the control (Fig. 5B). After fluorescence microscopy cells were analyzed for GABARAPs expression via western blotting. A significant decrease of GABARAPs expression after specific knockdown was verified when compared with that of the control and with cells that were transfected with scrambled siRNA (Supplementary Figure 8A and B).

## Discussion

The role of autophagy in degradation and immunoregulation implicates this process as a critical target for HIV in the maintenance of viral persistence and immune evasion. Therefore, HIV has evolved strategies to inhibit autophagy effectively and use it for its own benefit^60, 35^. For example, Nef induces autophagy but simultaneously inhibits the autophagosome-lysosome fusion via its association with Beclin 1^61, 62^. This process is not only important for progression to clinical AIDS but also releases autophagic proteins and enables these proteins to function in alternative pathways.

In the present study, we show that Nef immunoprecipitates (Fig. 1) and directly interacts (Fig. 2A) with GABARAP, GABARAPL1 and GABARAPL2, but not with the LC3 family member LC3B. LC3B can be converted into a Nef binding protein by replacement of only the conserved residues S53 and F62 of GABARAP family members (Fig. 3D). We further show that Nef colocalizes with GABARAPs at the plasma membrane and at vesicular structures within the cytoplasm (Fig. 4). Under *in vivo* conditions co- and posttranslational modifications occur, namely N-myristoylation of Nef^63^ and covalent phosphatidylethanolamine attachment for GABARAP^19^, both of which enhance membrane association of Nef and GABARAP, increase their local concentration and thus promote complex formation between Nef and GABARAP as seen in the colocalization experiments. Finally, GABARAPs-targeted siRNAs effectively reduce the plasma membrane localization of Nef (Fig. 5). In particular the association of Nef with the inner leaflet of the plasma membrane and also with endocytic and perinuclear vesicles is essential for most of the activities of Nef^17^. Approximately 10 to 30% of total Nef is membrane-associated at equilibrium^9, 12–16^. In a recent study, the N-terminal domain of Nef was replaced with other heterologous membrane targeting domains to investigate the respective subcellular localization of Nef variants. Surprisingly, this study revealed that the activities of Nef are independent of the strength of Nef membrane association, the steady state subcellular localization of Nef, and the anterograde transport pathway exploited by Nef, but critically depend on dynamic vesicular transport of Nef to the plasma membrane. Thus, the conclusion drawn from this research is that the plasma membrane transport itself regulates the biological activity of Nef. Currently the underlying transport mechanism of Nef is poorly defined, but the involvement of small transport vesicles has been suggested^17^.

Here, we show for the first time that the localization of Nef at the plasma membrane is strongly dependent on GABARAPs. Previous studies have clearly revealed the necessity of GABARAPs for the modulation of membrane expression of different receptor proteins at the plasma membrane^29–33, 64, 65^ by acting as a cargo receptor that incorporate these receptors to transport vesicles. Nef, however, is the first protein of such kind that is not an integral transmembrane protein.

We observed colocalization of Nef and GABARAPs in small vesicular structures throughout the cytoplasm and the plasma membrane that could possibly represent those transport vesicles. GABARAP family members have already been described to facilitate transport of vesicular structures along microtubules^54, 55^ to the plasma membrane and to mediate the fusion of these vesicles with the plasma membrane^56–58^.

In summary we identified GABARAP, GABARAPL1 and GABARAPL2 as the first direct interaction partners of Nef that are essential for its plasma membrane localization. As the recruitment of Nef to the plasma membrane is fundamental for Nef-mediated pathogenesis, the inhibition of the GABARAP-Nef interaction may represent a therapeutic strategy.

## Methods

### Recombinant protein preparation

Plasmid construction, expression, and purification of human GABARAP, GABARAPL1, GABARAPL2 and LC3B in *Escherichia coli (E.coli)* BL21 DE3 have been described previously^40, 66^. GABARAP (S53D/F62L) and LC3B(D56S/L65F) mutants were generated by QuikChange II Site-Directed mutagenesis (AgilentTechnologies, Böblingen, Germany) using oligonucleotides 5′-GAAATACCTGGTGCCTGATGATCTCACAGTTGGTCAGTTCTAC AAGTTGATCCGGAAGC-3′ and 5′-GCTTCCGGATCAACTTGTAGAACTGACCAACT GTGAGATCATCAGGCACCAGGTATTTC-3′ for GABARAP and 5′- GTTCCTTGTAC CTTCCCATGTCAACATGAGTGAGCTCATCTTCATAATTAGAAGGCGC-3′ and 5′-GC GCCTTCTAATTATGAAGATGAGCTCACTCATGTTGACATGGGAAGGTACAAGGAAC-3′ for LC3B, respectively.

A Nef gene (SF2) optimized for *E.coli* expression and extended C-terminally by a hexahistidine (His)-tag was purchased as a synthetic gene supplied in a standard vector (Geneart, Invitrogen), and was subcloned into a modified pETDuet expression vector (pETDuet-1Δ6His_Nef97)^66^, hereby substituting the original viral Nef gene, using NdeI and XhoI restriction sites, yielding the expression plasmid pETDuet_MCS2_codon_opt_Nef_HisTag. Expression and purification of Nef was performed in *E.coli* BL21(DE3)T1* according to a published procedure^66^. Purity of the recombinant proteins has been judged by SDS-PAGE analysis and Coomassie brilliant blue (CBB) staining (Supplementary Fig. 3).

### Eukaryotic plasmids

The plasmid pDsRed-N2/Nef coding for Nef N-terminally fused to DsRed has been described elsewhere^34^. Plasmid peCFP-N1/Nef was generated by subcloning the PCR-amplified Nef coding region from pDsRed-N2/Nef into restriction sites BamHI and HindIII of peCFP-N1 (Clontech). Genes for GABARAP, GABARAPL1, GABARAPL2 and LC3B were subcloned from described GST-fusion plasmids by PCR amplification into the XhoI and BamHI sites of peYFP-C1 (Clontech), yielding peYFP-C1/GABARAP, peYFP-C1/GABARAPL1 and peYFP-C1/GABARAPL2. Sequences of all constructs were confirmed by sequencing.

### Antibodies and chemicals

Rabbit polyclonal anti-GFP also reacting with EYFP, ECFP (sc-8334) and mouse monoclonal anti-DsRed2 (sc-101526) were obtained from Santa Cruz Biotechnology, Inc. (Dallas, TX, USA). Rabbit polyclonal antibodies raised against GABARAP (18723-1-AP), GABARAPL2 (18724-1-AP), GABARAPL1 (18721- 1-AP), and LC3B (18725-1-AP) were obtained from Proteintech Group, Inc. (Chicago, IL, USA). The loading control antibody was the rabbit polyclonal anti-tubulin antibody (ab59680, Abcam, Cambridge, UK) whereas the horseradish peroxidase (HRP) conjugated secondary antibody from donkey raised against mouse IgG (sc-2020) and from goat raised against rabbit IgG (sc-2030) were from Santa Cruz Biotechnology.

### Cell culture

The HEK293 (Human embryonic kidney 293) cell line was obtained from DSMZ (Braunschweig, Germany) and cultivated in Dulbecco’s modified Eagle’s medium (DMEM, GE Healthcare) with 4.5 g/l glucose containing 10% fetal bovine serum at 37 °C and in a 5% CO_2_ atmosphere. Cells were subcultivated and counted using a cover slipped improved Neubauer chamber at confluence between 80 and 90%. Cells were routinely passaged with Trypsin/EDTA at a constant 1:10 split ratio and used between passages 10 and 30 for all experiments. Cells were regularly tested for mycoplasma contamination.

For cell lysis cells were rinsed with ice cold PBS, and approximately 1 × 10^7^ cells were incubated with 1 ml ice-cold lysis buffer (150 mM NaCl, 1% Triton-X-100, 0.5% sodium deoxycholate, 0.1% SDS, 50 mM Tris, pH 8.0) without sonication, followed by centrifugation at 12,000 *g* for 30 min at 4 °C. The total protein content of the supernatants was calculated with a BCA assay (BioRad).

### Transfection

For transient expression experiments, HEK293 cells (1 × 10^5^ cells/well) were seeded in six-well dishes. Transfections were performed with 2 μg of total DNA using Polyfect (Qiagen, Hilden, Germany), according to the manufacturer’s instructions. The medium was replaced 4 h after transfection with DMEM including 10% fetal bovine serum (FBS), 2 mM glutamine and 1 mM sodium pyruvate. For stable expression experiments, HEK293 cells were transfected as described above. Two days after transfection, cells were cultured in DMEM supplemented with FBS (10% v/v) and G418 (1 mg/ml) (Applichem, Chicago, IL, USA) for about 4 to 6 weeks for selection. Ongoing cultures were grown in the absence of antibiotics.

### Pull-down assays

For direct binding assays 50 μM of purified recombinant Nef was immobilized on 50 μl NHS-activated Sepharose 4 Fast Flow (Amersham Pharmacia Biotech) according to the manufacturer’s instructions. After incubation of 25 μM of the different purified recombinant ATG8s or their respective mutant proteins in 200 μl binding buffer with Nef-coupled or uncoupled Sepharose for 4 h at 4 °C, the flow through was collected, the Sepharose beads were washed three times with 250 μl of 1x PBS, and pulled down proteins were eluted with 200 μl of elution buffer (50 mM glycine, pH 2.3). Following precipitation with methanol/chloroform, flow through, wash and eluate fractions were analyzed by SDS-PAGE and CBB staining.

### Immunoprecipitation assay

For immunoprecipitation from HEK293 cell lysates, antibodies (as outlined above) were covalently coupled to amino reactive agarose beads (Pierce Co-IP Kit, Thermo Scientific), following the manufacturer’s instructions. In each experiment, 500 μl of cell lysate supernatant was applied on a spin column filled with the appropriate antibody-coupled agarose beads or filled with un-coupled control beads. After washing three times with 200 μl of lysis buffer, bound protein was recovered after incubation with 60 μl of elution buffer at room temperature. Following precipitation with methanol/chloroform, proteins from the flow through fractions, the eluates as well as appropriate input controls were analyzed by SDS-PAGE and western blotting, using antibodies as outlined in the text.

### SDS-PAGE

Protein samples were subjected to denaturing electrophoresis on 15% and any-kd gradient (Biorad, Munich, Germany) sodium dodecyl sulfate-polyacrylamide gels. Proteins were stained with CBB.

### Western blotting

Proteins were transferred from the gel to a polyvinylidene difluoride membrane (GE Healthcare) which was blocked with 5% milk powder in TBS-T (Sigma-Aldrich) for 60 min. The membrane was incubated with a primary antibody overnight, followed by incubation with the peroxidase conjugated secondary antibody for 60 min. Blots were visualized by chemiluminescence (SuperSignal West Dura Chemiluminescent Substrate, Pierce) and documented using the ChemiDoc system (Bio-Rad).

### Live cell imaging

Cells were grown on glass bottom wells (MatTek, Ashland, MA, USA), and incubated under the conditions described above. During live cell imaging cells were incubated in a PeCon incubation chamber (Erbach, Germany) at 37 °C and 5% CO_2_. Images were recorded with an LSM 710 confocal laser scanning system (Carl Zeiss Microimaging, Jena) using a Plan-Apochromat 63 ×/1.40 Oil DiC objective.

### RNA interference

For siRNA silencing subconfluent HEK293 cells were transfected with different pools of siRNA (50 nM from each siRNA SMARTpool) using DharmaFect 1 (Dharmacon, Lafayette, CO, USA) following the manufacturer’s instructions. siRNA SMARTpools targeting GABARAP (M-012368-01), GABARAPL1 (M-014715-01) or GABARAPL2 (M-006853-02) consisting of four RNA duplexes each and a non-targeting siRNAs control pool (D-001206-14) were purchased from Dharmacon.

### NMR spectroscopy

The Nef binding interface on GABARAP was determined by an NMR titration experiment. NMR spectra were recorded using a cryogenically cooled *Z*-pulse field gradient ^1^H[^13^C,^15^N] probe at 25 °C on a Varian INOVA NMR system at a proton frequency of 600 MHz. The initial sample contained 200 µM ^15^N-GABARAP in 25 mM NaH_2_PO_4_ (pH 6.9), 100 mM KCl, 100 mM NaCl, 50 μM EDTA, 5 mM β-mercaptoethanol and 7% (v/v) deuterium oxide (D_2_O). Successive addition of unlabeled Nef (stock solution: 120 µM) resulted in samples containing protein ratios of 1:0.5 (90 µM GABARAP: 45 µM Nef), 1:1 (60 µM GABARAP: 60 µM Nef) and 1:1.5 (48 µM GABARAP: 72 µM Nef), respectively. Self-aggregation of Nef at concentrations >120 µM prohibited titration steps at higher concentrations. Thus, saturation of the system was not achieved and prohibited determination of the dissociation constant. 2D ^1^H-^15^N-HSQC spectra were collected with 150 complex points in the ^15^N time domain, up to 384 scans per *t*1 point, and a 2 s recycle delay. Data were processed with NMRPipe^67^ and analyzed with CcpNMR^68^. The weighted chemical shift changes upon interaction were calculated using the following equation:

$${\rm{\Delta }}\delta ({}^{1}H,{}^{15}N)=\sqrt{{\rm{\Delta }}\delta {({}^{1}H)}^{2}+{(\frac{{\rm{\Delta }}\delta ({}^{15}N)}{5})}^{2}}$$

To prove structural integrity of the mutants GABARAP (S53D/F62K) and LC3B(D56S/K65F), ^15^N-labeled samples of these proteins were prepared. 2D ^1^H-^15^N-HSQC spectra were recorded and processed as described above.

### Databases and software

The following entries for the ATG8 protein sequences were used (GABARAP: *O95166;* GABARAPL1: *Q9H0R8*; GABARAPL2: *P6052*0; LC3A: *Q9H49*2; LC3B: *Q9GZQ8*; LC3B2: *A6NCE7*; LC3C: *Q9BXW4*) and have been retrieved from the UniProtKB database^69^. Protein sequence alignments have been created using Clustal Omega alignment tools^70^. Structure representations were created with PyMOL (The PyMOL Molecular Graphics System, Version 1.7.1.3, Schrödinger, LLC). Coordinates were downloaded from the RCSB Protein Data Bank (http://www.rcsb.org/pdb/). Figures were arranged using CorelDRAW Graphics Suite X6 (Corel Corporation).

### Statistics

Data were analyzed using two-tailed unpaired Student’s t-tests. All experiments were performed at least three times, with data representing mean values ± s.d. (standard deviation).

## Electronic supplementary material

Supplementary Information
