# Supplementary material for: Direct binding to GABARAP family members is essential for HIV-1 Nef plasma membrane localization
Source: Sci Rep. 2017 Jul 20;7:5979. doi: 10.1038/s41598-017-06319-4 (PMC5519724; doi:10.1038/s41598-017-06319-4)
Supplement: Supplementary file 1 — Supplementary Information [file 41598_2017_6319_MOESM1_ESM.pdf]

Direct binding to GABARAP family members is essential for HIV-1 Nef plasma membrane localization

Alexandra Boeske[1,2], Melanie Schwarten[1], Peixiang Ma[1#], Markus Tusche[1], Jessica Mötter[1], Christina Möller[1,2], Philipp Neudecker[1,2], Silke Hoffmann[1§], Dieter Willbold[1,2§]

[1]Institute of Complex Systems, Structural Biochemistry (ICS-6), Forschungszentrum Jülich, 52425 Jülich, Germany

[2]Institut für Physikalische Biologie, Heinrich-Heine-Universität Düsseldorf, 40225 Düsseldorf, Germany

[#] Present address: Shanghai Institute for Advanced Chemical Studies (SIAIS)
ShanghaiTech University, Shanghai, 201012, China

[§]To whom correspondence may be addressed:
Dieter Willbold d.willbold@fz-juelich.de
Silke Hoffmann si.hoffmann@fz-juelich.de

Supplementary Figure 1 is related to Figure 1A-C
Supplementary Figure 2 is related to Figure 1D-F
Supplementary Figure 3 is related to Figure 2A and 3D
Supplementary Figure 4 is related to Figure 2A
Supplementary Figure 5 is related to Figure 2B
Supplementary Figure 6 is related to Figure 3
Supplementary Figure 7 is related to Figure 3D
Supplementary Figure 8 is related to Figure 5B

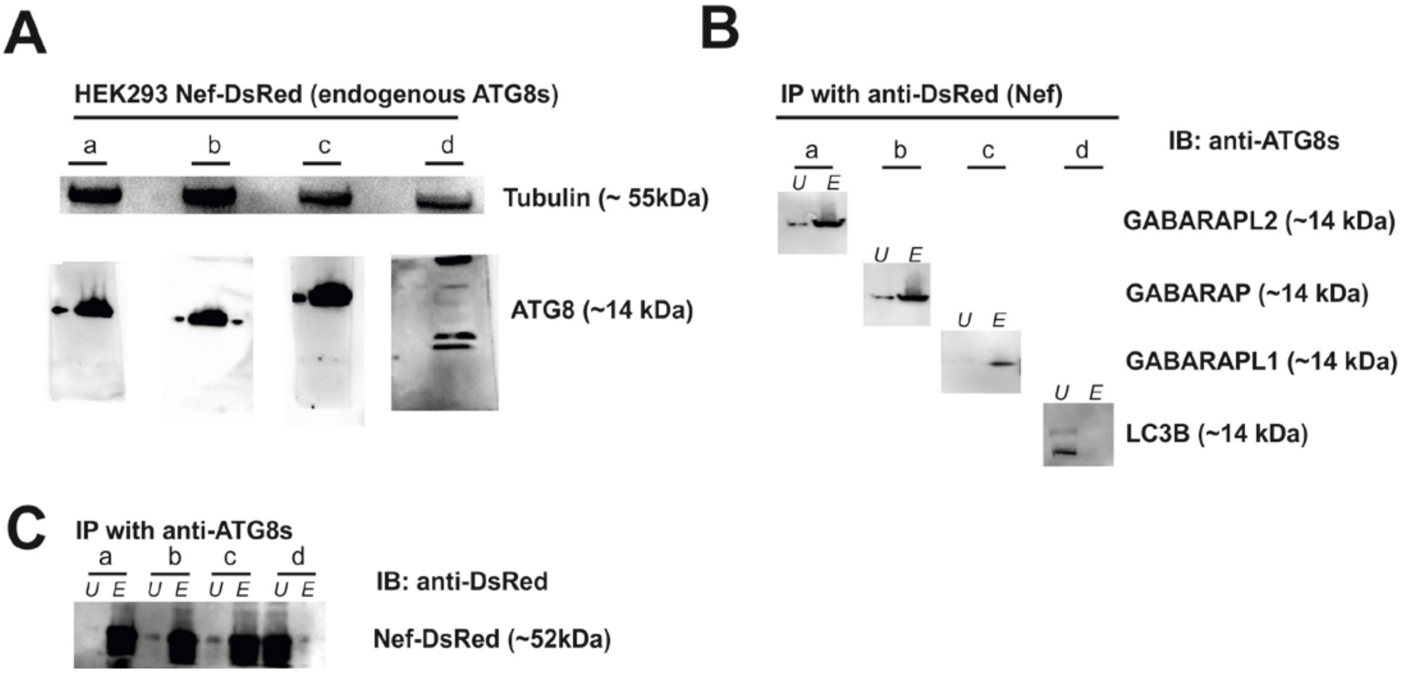

Supplementary Figure 1: Co-immunoprecipitation (IP) of the Nef-ATG8 complex. Full length blots of co-immunoprecipitation studies with lysates prepared from HEK293 cells stably expressing Nef-DsRed **(A-C)**. **(A)** shows samples before immunoprecipitation. In **(A)** an anti-GABARAPL2 (a), an anti-GABARAP (b), an anti-GABARAPL1 (c) and an anti-LC3B antibody (d) were used to detect the different ATG8s. **(B)** Immunoprecipitates with anti-DsRed antibody followed by SDS-PAGE and immunoblotting with various ATG8 antibodies (a: anti-GABARAPL2, b: anti-GABARAP, c: anti-GABARAPL1 and d: anti-LC3B). **(C)** In a reciprocal set of experiments, immunoprecipitates were carried out with various ATG8 (a: anti-GABARAPL2, b: anti-GABARAP, c: anti-GABARAPL1 and d: anti-LC3B) antibodies followed by SDS-PAGE and immunoblotting with an anti-DsRed antibody. (U: unbound material; E: eluate fraction).

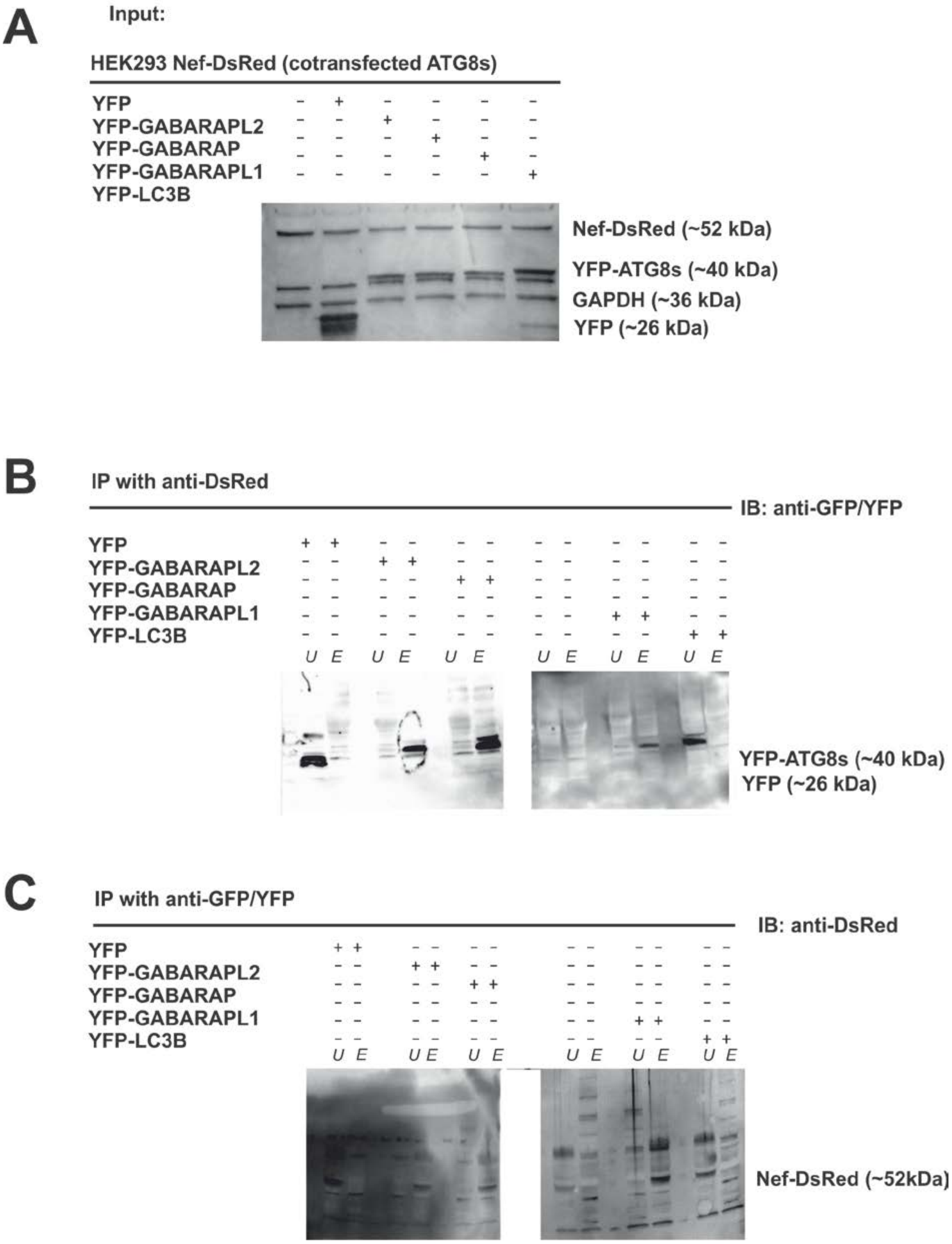

Supplementary Figure 2: Full length blots of Co-immunoprecipitation (IP) of the Nef-ATG8 complex. In **(A)** an anti-DsRed antibody was used to detect Nef fused to DsRed and an anti-YFP antibody was used to detect YFP and the ATG8s fused to YFP. **(B)** Immunoprecipitates with an anti-DsRed antibody or **(C)** with an anti-GFP antibody followed by SDS-PAGE and immunoblotting with the antibodies are indicated. Data are representative of three independent experiments (U: unbound material; E: eluate fraction).

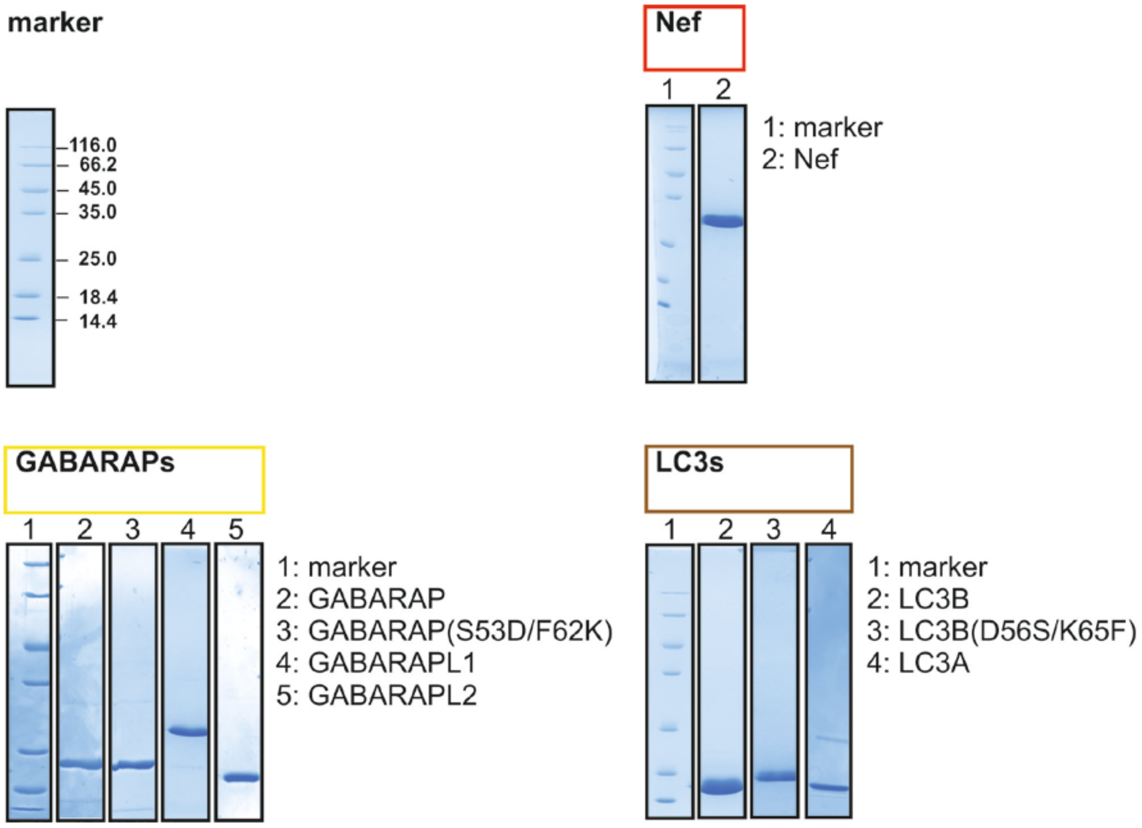

Supplementary Figure 3: Purity of the recombinant proteins GABARAP, GABARAPL1, GABARAPL2, LC3B, HIV-1 Nef and their mutants. Recombinant proteins were analyzed by 12% or 15% SDS-PAGE. CBB staining demonstrates high purities of the recombinant proteins used throughout this study (molecular weight standard: SM0431, Pierce).

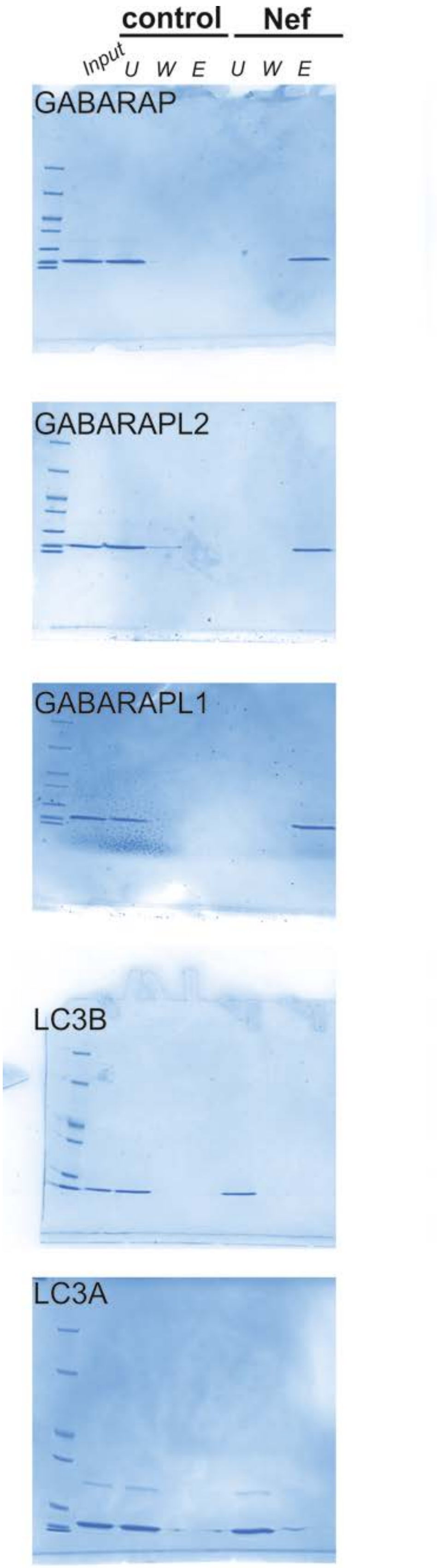

Supplementary Figure 4: Nef selectively binds to GABARAPs in a direct manner and contacts the canonical ligand binding site of GABARAP. Full length images of pull-down assays. Nef-conjugated or free control Sepharose beads were incubated with the purified recombinant ATG8 paralogs listed. The input, the unbound material of the flow through (U), the wash (W) fractions and the eluate (E) fractions have been analyzed by SDS-PAGE and CBB staining.

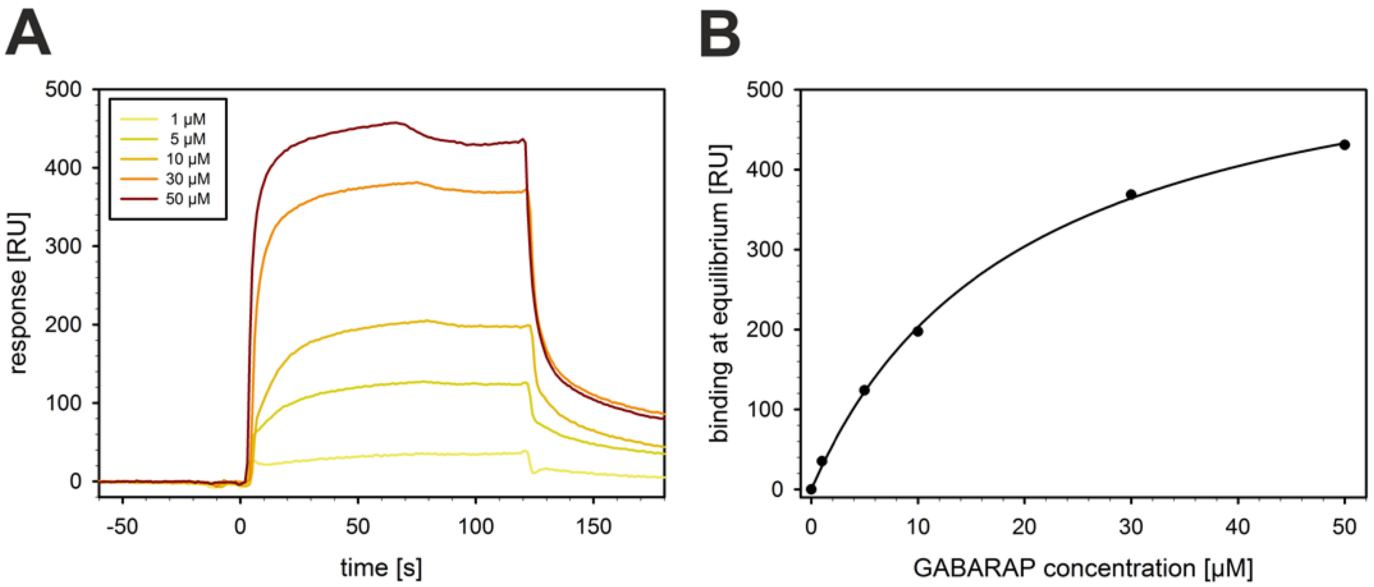

Supplementary Figure 5: Binding of GABARAP to Nef investigated by surface plasmon resonance spectroscopy (SPR). (A) Sensorgram resulting from injections of GABARAP at different concentrations with Nef immobilized on the chip and measured on a BiacoreX optical biosensor (GE Healthcare). Following standard procedures for amine coupling, 30 µM of recombinant Nef without the fusion tag in 10 mM sodium acetate, pH 4.5, was used for coupling to the carboxymethylated dextran matrix of a CM5 sensor chip surface. A reference surface was treated identically, but not exposed to Nef for immobilization. Recombinant GABARAP was dissolved in running buffer (10 mM 4-(2-hydroxyethyl)-1-piperazineethanesulfonic acid (HEPES), pH 7.4, 150 mM NaCl, 3 mM EDTA, 1 mM β-mercaptoethanol, and 0.005% surfactant P20) at concentrations between 1 and 50 µM and injected at 20 µl/min and 25°C. Response units (RUs) were recorded as a function of time. (B) Steady state binding fit of GABARAP interacting with immobilized Nef. The averaged RUs between 105 and 115 s after injection were used for evaluation. Nonlinear regression analysis according to the steady state affinity model yielded a K_d for GABARAP-Nef of 19.7 ± 1.1 µM.

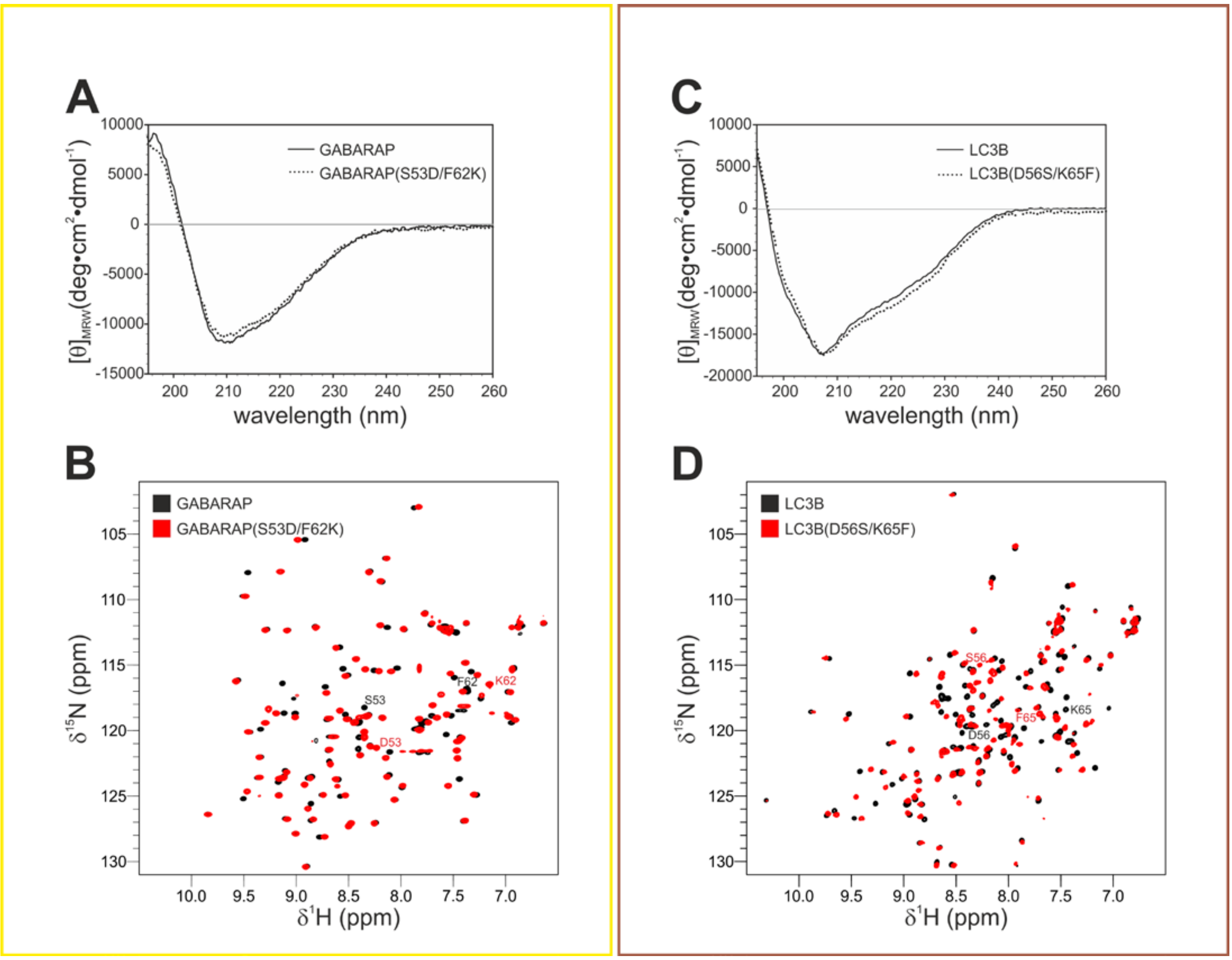

Supplementary Figure 6: Structural integrity of GABARAP and LC3B is maintained in the GABARAP (S53D/F62K) and C3B (D56S/K65F) mutant proteins. Protein secondary structure was analyzed by far-UV CD spectroscopy. CD spectra of wild-type GABARAP (A) and wild-type LC3B (C) and the mutant proteins were recorded on a Jasco J810 spectrometer (Jasco, Groß-Umstadt, Germany) at 298 K in a 0.1 cm path-length quartz cuvette (Hellma, Müllheim, Germany) at a protein concentration of 7 μM based on UV light absorption at 280 nm. Data collection was performed between 260 and 185 nm with 0.5 nm increments and a bandwidth of 1 nm. Spectra were baseline-corrected (SigmaPlot 11; Systat Software, Inc.). Units are given as the mean residue ellipticity ($[\theta]_{MRW}$). Clearly, wild-type and mutant proteins show nearly identical spectra, suggesting that the conformation of the mutants is similar to the wild-type proteins. The overall structural integrity of GABARAP (B) and LC3B (D) wild-type and the mutant proteins was further analyzed by NMR. 2D ^{1}H-^{15}N-HSQC-spectra overlay of the wild-type (black) and mutant (red) proteins show chemical shift changes only for the mutated residues and residues in close proximity to the mutation, indicating that the overall fold is not affected by the mutations.

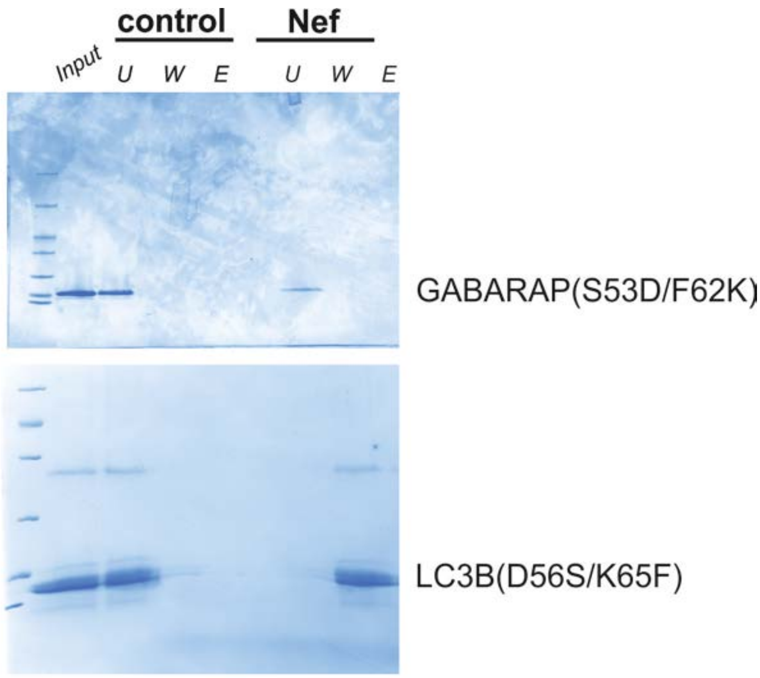

Supplementary Figure 7: GABARAP residues S53 and S62 are essential for Nef binding. Full length images of pull-down assays. Nef-conjugated or free Sepharose beads (control) were incubated with GABARAP(S53D/F62K) or LC3B(D56S/K65F) proteins. The input, the unbound material of the flow through (U), the wash (W) fractions and the eluate (E) fractions were subjected to SDS-PAGE and visualized by CBB staining.

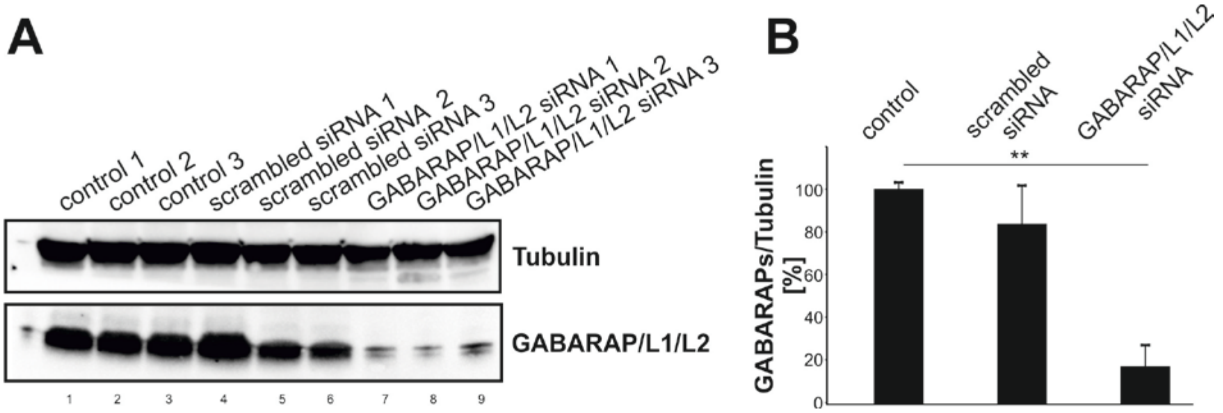

Supplementary Figure 8: Efficiency of GABARAPs knockdown

(A) HEK293 cells stably expressing Nef-DsRed and mock transfected (control), transfected with scrambled siRNA or with a pool of GABARAP, -L1 and –L2 siRNAs, presented in Fig. 5, were collected after live-cell imaging and analyzed for GABARAPs expression via western blot analysis. In this case, tubulin was used as the loading control. **(B)** Quantification of GABARAPs expression levels after treatment with the respective siRNAs. Western blots for quantification were detected with a ChemiDoc MP (Bio-Rad) and the signal strength was analyzed with Image Lab. To allow comparison between different samples, all signal strengths were normalized to the signal strength of tubulin. The average value of three control experiments was set to 100%.
